# Structural and antigenic characterization of a novel genotype of Mfa1 fimbriae in *Porphyromonas gingivalis*

**DOI:** 10.1080/20002297.2023.2215551

**Published:** 2023-05-21

**Authors:** Miyuna Fujimoto, Yoshikazu Naiki, Kotaro Sakae, Tomohiko Iwase, Naoyoshi Miwa, Keiji Nagano, Hiroyuki Nawa, Yoshiaki Hasegawa

**Affiliations:** a Department of Microbiology, School of Dentistry, Aichi Gakuin University, Nagoya, Japan; b Department of Pediatric Dentistry, School of Dentistry, Aichi Gakuin University, Nagoya, Japan; c Division of Microbiology, Department of Oral Biology, School of Dentistry, Health Sciences University of Hokkaido, Hokkaido, Japan

**Keywords:** *Porphyromonas gingivalis*, Mfa1, fimbriae, periodontitis, genotype

## Abstract

**Background:**

Mfa1 fimbriae of the periodontal pathogen Porphyromonas gingivalis are responsible for biofilm formation and comprise five proteins: Mfa1–5. Two major genotypes, mfa1^70^ and mfa1^53^, encode major fimbrillin. The mfa1^70^ genotype is further divided into the mfa1^70A^ and mfa1^70B^ subtypes. The properties of the novel mfa1^70B^ remain unclear.

**Methods:**

Fimbriae were purified from P. gingivalis strains JI-1 (mfa1^70A^), 1439 (mfa1^70B^), and Ando (mfa1^53^), and their components and their structures were analyzed. Protein expression and variability in the antigenic specificity of fimbrillins were compared using Coomassie staining and western blotting using polyclonal antibodies against Mfa1^70A^, Mfa1^70B^, and Mfa1^53^ proteins. Cell surface expression levels of fimbriae were analyzed by filtration enzyme-linked immunosorbent assays.

**Results:**

The composition and structures of the purified Mfa1 fimbriae of 1439 was similar to that of JI-1. However, each Mfa1 protein of differential subtype/genotype was specifically detected by western blotting. Mfa1^70B^ fimbriae were expressed in several strains such as 1439, JKG9, B42, 1436, and Kyudai-3. Differential protein expression and antigenic heterogeneities were detected in Mfa2–5 between strains.

**Conclusion:**

Mfa1 fimbriae from the mfa170A and mfa170B genotypes indicated an antigenic difference suggesting the mfa170B, is to be utilized for the novel classification of P. gingivalis.

## Introduction

The gram-negative anaerobe *Porphyromonas gingivalis* is associated with the development of periodontal disease [1]. Although it is only present in small numbers, *P. gingivalis* may be a keystone pathogen that subverts host innate immunity and induces dysbiosis and chronic inflammation [2,3]. This organism may also contribute to systemic disorders, such as premature birth and the development of atherosclerosis, rheumatoid arthritis, diabetes mellitus, and Alzheimer’s disease [4–7].

*P. gingivalis* expresses various virulence factors, including proteases (gingipains), lipopolysaccharides, and most notably, fimbriae [8], which form a multi-species biofilm that colonizes periodontal tissues [9]. At least two distinct types of fimbriae are expressed in the bacterium, namely FimA and Mfa1 fimbriae [9]. The Mfa1 fimbria mainly comprises Mfa1 protein polymers encoded by *mfa1* in the *mfa* gene cluster [9]. Mature fimbriae also contain the minor proteins Mfa2–5, encoded downstream of *mfa1* (Figure S1) [10]. Mfa2 is localized in the basal portion of the structure and functions as an anchor and elongation terminator [11,12]. We previously reported that Mfa3 and Mfa4 were detected in purified fimbriae as 40 and 30 kDa bands, respectively, in a sodium dodecyl sulfate-polyacrylamide gel electrophoresis (SDS-PAGE) gel, and Mfa5 was detected as two bands, 130 and 150 kDa. Mfa3, Mfa4, and Mfa5 participate in the assembly of accessory protein complexes on the tips of fimbriae [13–15]. Recent structural and mechanistic analyses of the fimbrial proteins of *P. gingivalis* have revealed that the Mfa1–4 proteins are polymerized by a proteinase-mediated donor strand exchange mechanism, which is classified as a novel type of fimbriae designated as type V fimbriae [16,17]. The precursor of Mfa1 in *P. gingivalis*, the ATCC 33,277 strain, is cleaved for maturation at Arg^49^, located at the N-terminus via gingipains [18]. Additionally, Mfa1 fimbriae have recently been reported to modulate innate immune responses [19,20].

The *fimA* gene, encoding FimA, a major fimbrillin of the FimA fimbriae, is classified into five genotypes (I – V) [21,22]. FimA fimbriae present variability in antigenic specificity among genotypes I – V [23,24]. Studies from several countries have reported that genotypes II and IV are predominantly detected in patients with severe periodontitis, whereas genotype I is prevalent in healthy individuals or patients with mild periodontitis [25–27]. However, genotype I has also been detected at a high frequency in patients with severe periodontitis [28,29]. The discrepancies between these results indicate that the pathogenic diversity of *P. gingivalis* cannot be explained by the *fimA* genotype alone.

In contrast, we previously reported two genotypes of the *mfa1* gene, which encodes the major protein of Mfa1 fimbriae. We named them *mfa1*^*70*^ (70 kDa) and *mfa1*^*53*^ (53 kDa) based on the apparent molecular weights of the Mfa1 proteins encoded by the genes [30]. Moreover, we have recently analyzed the *mfa1* genotype in 12 uncategorized *P. gingivalis* strains by next-generation sequencing and used published genomic information on over 70 *P. gingivalis* strains to investigate the polymorphisms of all genes in the *mfa* gene cluster. We discovered that the *mfa1*^*70*^ genotype was further divided into two subtypes: *mfa1*^*70A*^ and *mfa1*^*70B*^ (Figure S2) [31]. Most *P. gingivalis* strains, including genome-sequenced ATCC 33,277, W83, and TDC60 strains, have been classified as subtype *mfa1*^*70A*^, and uncategorized strains such as 1436, 1439, B42, JIKG9, and Kyudai-3 have been classified as the new subtype *mfa1*^*70B*^ (Table 1). However, the properties of subtype *mfa1*^*70B*^ fimbriae and the relationship between genotypes/subtypes, pathogenesis, and variability in antigenic specificity have not been studied [31,32]. Moreover, *mfa2–5* genotypes do not necessarily correlate with *mfa1* genotypes. The *mfa2*, *mfa3*, and *mfa4* genes are divided into two genotypes: 70 and 53 [31], and *mfa5* can be genotyped independently of *mfa1–4* as A – E genotypes (Figure S2). Therefore, these sequence differences among the strains of genotypes of fimbrial proteins are indicative of antigenic variation. The aim of this study was to characterize subtype *mfa1*^*70B*^ fimbriae by comparing the structure, components, antigenicity, and expression of fimbriae on the cell surface. Furthermore, the protein expression and antigenicity of Mfa2, Mfa3, Mfa4, and Mfa5 in *P. gingivalis* strains were investigated.

**Table 1.** Porphyromonas gingivalis strains and genotypes, and protein expression of mfa1–mfa5.

Strain	*mfa1*	*mfa2*	*mfa3*	*mfa4*	*mfa5–1*	*mfa5–2*
ATCC 33,277	70A	70	70	70	A1	
EM3	70A	70	70	70	X	X
D83T3	70A	70	70	70	A2	E
TV14	70A(M)	70	70	70	D	
1439	70B	70	70	70	A2	
JKG9	70B	70	70	70	A2	
B42	70B	70	70	70	A2	
1436	70B	70	70	70	A2	
Kyudai-3	70B	70	70	70	A2	E
Ando	53	53	53	53	X	
B158	X	70	70	70	A2	
JKG10	X	70	70	70	A2	X
Kyudai-4	X	70	70	70	A2	
222	-	-	-	-	X	X
JI-1	*fimA* mutant derived from 33,277 [11]					
SMF-1	*mfa1* mutant derived from 33,277 [33]					

-, no corresponding gene was detected.

X, unknown.

Highlights in dark and light gray indicate strong and weak signals, respectively, as detected by western blotting.

M, a possible point mutation, such as a nonsense mutation, was detected.

## Materials and methods

### *P. gingivalis* strains and culture conditions

Twelve bacterial strains with previously analyzed draft genomes [31] were used in this study and are summarized in Table 1. We used a *P. gingivalis* JI-1 *fimA* mutant derived from ATCC 33,277, with subtype *mfa1*^*70A*^ [11], *P. gingivalis* 1439 with subtype *mfa1*^*70B*^ [24], and *P. gingivalis* Ando with genotype *mfa1*^*53*^ [30], as prototype strains for the purification of fimbriae. Moreover, the *P. gingivalis* SMF1 *mfa1* mutant derived from *P. gingivalis* ATCC 33,277 [33] was used as the negative control for enzyme-linked immunosorbent assay (ELISA). *P. gingivalis* was maintained on Brucella HK agar (Kyokuto Pharmaceutical Industrial Co., Ltd., Tokyo, Japan) supplemented with 5% laked rabbit blood at 37°C under anaerobic conditions. Liquid cultures were grown in trypticase soy broth (BD BBL) supplemented with 0.25% (w/v) yeast extract, 2.5 μg/mL hemin, 5 μg/mL menadione, and 0.1 μg/mL dithiothreitol (sTSB). When appropriate, the media were supplemented with 5 μg/mL chloramphenicol or 20 μg/mL erythromycin.

### Preparation of whole cell lysates (WCLs)

WCLs were prepared according to a standard protocol [34]. Briefly, *P. gingivalis* strains were cultivated at 37°C under anaerobic conditions in sTSB until they reached the early stationary phase. Bacterial cells were collected by centrifugation, resuspended in 10 mM HEPES-NaOH (pH 7.4) containing 0.1 mM *N*-α-p-tosyl-l-lysine chloromethyl ketone, 0.2 mM phenylmethylsulfonyl fluoride, and 0.1 mM leupeptin, and then lysed by a French press without sonication. Residual unbroken cells were removed by centrifugation at 1,000 ×*g* for 10 min at 4°C. The obtained supernatant was used as the WCL.

### Purification of fimbriae

Mfa1 fimbriae were purified as previously described [35]. Briefly, WCLs were mixed with ammonium sulfate at 50% saturation to precipitate the fraction containing fimbriae. Pure fimbriae were then obtained by fractionation twice using DEAE-sepharose fast flow chromatography (GE Healthcare Bio-Sciences AB, Uppsala, Sweden) with a linear gradient of NaCl (0–0.3 M).

### SDS-PAGE and immunoblot analysis

WCL and purified fimbriae were mixed with SDS and 2-mercaptoethanol and denatured by heating at 100°C for 5 min or 60°C for 10 min. After electrophoresis, the SDS gels were stained with Coomassie Brilliant Blue (CBB) or analyzed by western blotting. For western blotting, crude antisera containing a mix of polyclonal antibodies to Mfa1^53^ (see below), Mfa1^70A^ [15], Mfa1^70B^ (see below), Mfa2 (genotype 70), Mfa3 (genotype 70), Mfa4 (genotype 70), and Mfa5 (genotype A1) [11,13–15] were used as primary antisera. After reaction with a goat anti-rabbit horseradish peroxidase (HRP)-labeled secondary antiserum, the labeled proteins were developed using a chemiluminescence substrate (TakaraBio Inc., Kusatsu, Japan).

To raise antiserum against Mfa1^53^ and Mfa1^70B^, rabbits were immunized with purified Mfa1 fimbriae from *P. gingivalis* Ando and 1439 (Sigma-Aldrich, St. Louis, MO, USA), respectively.

### Protein structure prediction

SWISS-MODEL analysis was used for protein structure homology modeling based on the X-ray crystal structures deposited in the Protein Data Bank (PDB). X-ray crystal structures of the mature forms of the Mfa1 protein of ATCC 33,277 [36] have been previously published in the PDB database (5nf3.1.A). Structure-assisted multiple sequence alignment of Mfa1 proteins was performed using PROMALS3D [37].

### N-terminal amino acid sequence analysis

Pure Mfa1 fimbriae proteins from *P. gingivalis* 1439 separated by SDS-PAGE were then transferred to polyvinylidene difluoride membranes and stained with Ponceau S. Mfa1 bands were excised and analyzed by N-terminal sequencing using an ABI 477 A automatic peptide sequence analyzer at the Center for Instrumental Analysis (Hokkaido System Science Co., Ltd, Sapporo, Japan).

### Transmission electron microscopy

Pure fimbriae were placed onto carbon-coated 400-mesh copper grids, negatively stained with 2% uranyl acetate, and observed using an H-7600 transmission electron microscope at 100 kV (Hitachi, Chiyoda, Tokyo, Japan). One hundred fimbriae were randomly selected, and the fimbrial length was measured using electron microphotographs.

### Filtration ELISA

To detect Mfa1 fimbriae expressed on the cell surface, filtration ELISA was performed as previously described [13] using intact cells as the antigens. A 100 μL cell suspension containing 1 × 10^7^ cells was applied over filters in a 96-well MultiScreen-GV filtration plate with a pore size of 0.22 μm (MilliporeSigma, Burlington, MA, USA). Subsequently, the cells were washed with Tris-buffered saline containing 20 mM Tris pH 7.5, 150 mM NaCl, and 0.05% Tween 20 (TBST) and blocked with 3% bovine serum albumin in TBST. The cells were then probed with antiserum against each Mfa1 fimbriae type, washed, and labeled with HRP-conjugated polyclonal goat anti-rabbit IgG (Dako, Glostrup, Denmark). Subsequently, o-phenylenediamine and H_2_O_2_ in citrate buffer (pH 5.0) were added as the substrates. The reactions were terminated using 1 M H_2_SO_4_ and the absorbance at 490 nm (OD490) was measured. SMF1 was included as a negative control and the SMF1 value was subtracted from each measurement of the strains.

### Comparison of the amino acid sequences of Mfa proteins among P. gingivalis strains

Comparisons of the amino acid sequences of Mfa1–Mfa5 from various *P. gingivalis* strains were generated using the Clustal Omega multiple sequence alignment tool (https://www.ebi.ac.uk/Tools/msa/clustalo/).

### Statistical analysis

Data of filtration ELISA are expressed as means ± standard deviation (SD). One-way analysis of variance was performed, and results with *P*-values of <0.05 were considered significant. Differences between groups of fimbrial length were analyzed by the nonparametric Kruskal – Wallis test and were considered statistically significant at *P* < 0.05.

## Results

### Selection of prototype strain expressing Mfa1^70B^ fimbriae

We previously analyzed the draft genomes of 12 uncategorized strains of the *mfa1* genotype and reported that 3 of them were of the *mfa1*^*70A*^ genotype, 5 s were of the novel *mfa1*^*70B*^ genotype, and 4 were unknown (Table 1) [31]. In the present study, we confirmed Mfa1 expression in these strains. WCL from *P. gingivalis* were separated using SDS-PAGE and stained with CBB. The 70-kDa Mfa1 band [38] was clearly detected in the *mfa1*^*70A*^ strains ATCC 33,277, JI-1, and EM3 (Figure S3). Moreover, clear Mfa1 bands were detected in the *mfa1*^*70B*^ strains 1439, JKG9, and B42 at the same size as the Mfa1^70A^ bands. Among the above three strains, 43-kDa FimA bands [39] were detected in strains JKG9 and B42, but not in 1439. Contamination of other FimA fimbriae is a limitation in the structural and functional analyses of purified Mfa1 fimbriae. Therefore, we selected strain 1439, which detects the Mfa1 protein and not FimA, as the prototype strain for the purification of Mfa1^70B^ fimbriae. In contrast, the *mfa1*^*53*^ genotype Ando strain showed a 53-kDa Mfa1 band, but not a 70-kDa band [30]. No Mfa1 band was detected in the five uncategorized TV14, B158, JKG10, Kyudai-4, or 222 strains.

### Analysis of components of the purified Mfa1^70B^ fimbriae

**Figure 1.**
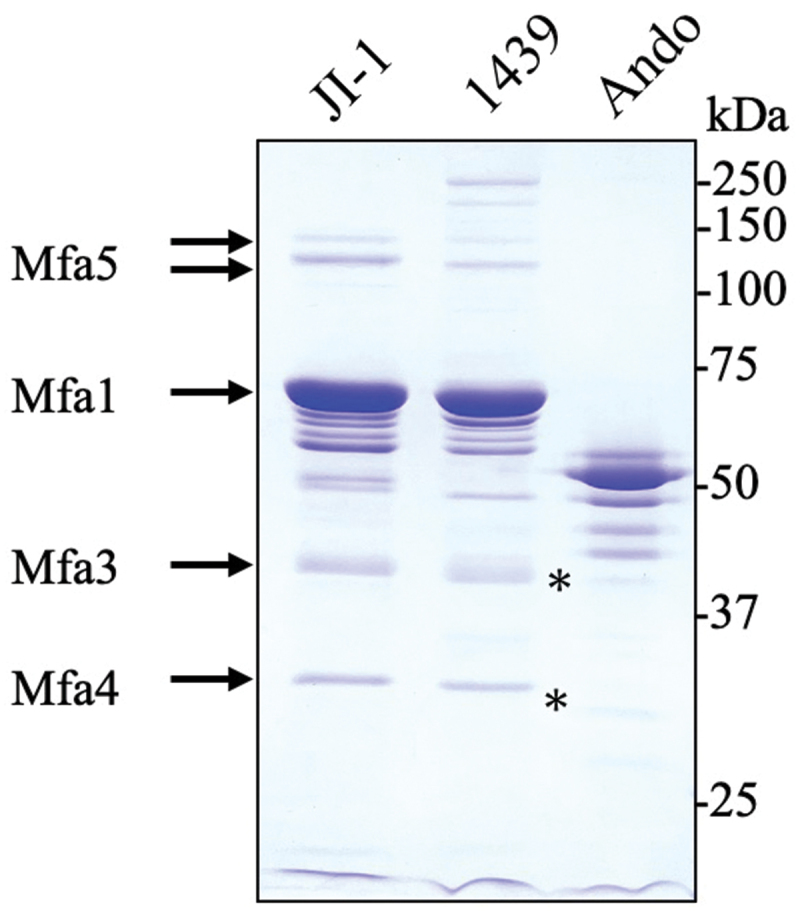
SDS-PAGE and CBB staining of the purified Mfa1 fimbriae of three prototype strains. Lanes were loaded with 3 µg of pure Mfa1 fimbriae. Arrows indicate the Mfa1, Mfa3, Mfa4, and Mfa5 bands of ATCC 33,277 [11]. The membrane protein Mfa2 was not detected in the purified fimbriae [11]. The weak bands of Mfa3 and Mfa4 detected in the Ando strain corresponding to Figure 7 are indicated by asterisks.

To compare the components and structures of the fimbriae, Mfa1 fimbriae of *P. gingivalis* JI-1, 1439, and Ando strains were purified. Initially, to confirm purification and analyze the components of the Mfa1 fimbriae, the fimbrial preparation was separated using SDS-PAGE and then stained with CBB. A distinct band at 70 kDa, the same size as that of the Mfa1^70A^ protein, was detected in the 1439 strain (Figure 1). In addition, three accessory proteins, 40-kDa Mfa3, 30-kDa Mfa4, and 130- and 150-kDa Mfa5 bands [13], were detected with the same band pattern as that of Mfa1^70A^ fimbriae. In contrast, a distinct band at 53 kDa was detected in the Ando strain; however, the bands corresponding to Mfa3–5 were weak (Figure 1).

**Figure 2.**
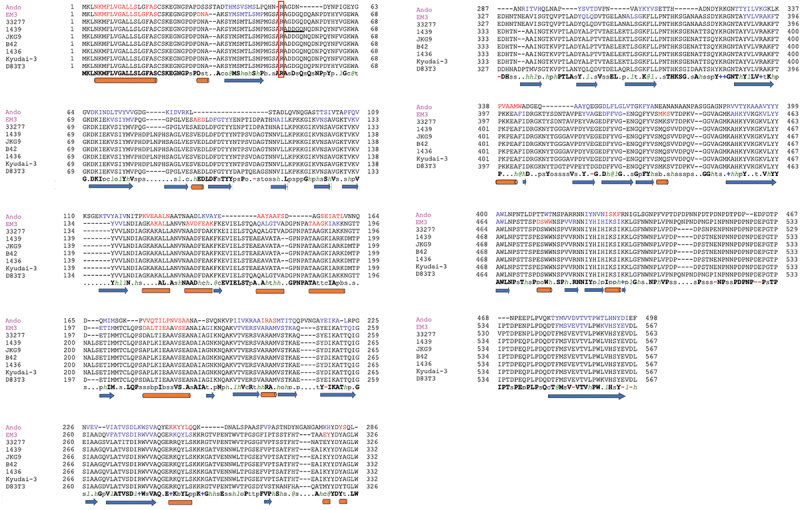
Structure-assisted multiple sequence alignment of Mfa1 proteins using PROMALS3D. the alignment is based on the precursor sequences of each Mfa1 protein (PDB code: 5dhm) structural model. The last line shows the consensus amino acid sequence. Representative sequences have magenta names and they are colored according to predicted secondary structures (red: alpha-helix, blue: beta-strand). Consensus secondary structure features are indicated as cylinders (α-helices) and arrows (β-strands) below the alignment. Consensus amino acid symbols are: conserved amino acids are in bold and uppercase letters; aliphatic (I, V, L): l; aromatic (Y, H, W, F): @; hydrophobic (W, F, Y, M, L, I, V, A, C, T, H): h; alcohol (S, T): o; polar residues (D, E, H, K, N, Q, R, S, T): p; tiny (A, G, C, S): t; small (A, G, C, S, V, N, D, T, P): s; bulky residues (E, F, I, K, L, M, Q, R, W, Y): b; positively charged (K, R, H): +; negatively charged (D, E): -; charged (D, E, K, R, H): c. the N-terminal amino acid sequence of 1439 is indicated by an underline. Amino acids within the gingipain cleavage site are highlighted by a red box. Upon cleavage by gingipain, the N-terminal region upstream of Arg^49^ is removed and polymerization is initiated.

Next, we analyzed the N-terminal amino acid sequence of the Mfa1^70B^ protein and identified the ADDGQ sequence, which precisely matched the sequence following the gingipain cleavage site at Arg^49^ (Figure 2; red box).

The protein structures of Mfa1 between different genotypes, including subtypes, were predicted and comparatively analyzed using SWISS-MODEL based on X-ray crystal structure data. The precursors of the amino acid sequences of Mfa1^70B^ (1439) and Mfa1^53^ (Ando) were aligned to those of Mfa1^70A^ (ATCC 33,277) using SWISS-MODEL. The structural model of the Mfa1^70B^ protein showed very high concordance with that of the Mfa1^70A^ protein (Figure S4); the Ν-terminal region containing the Arg^49^ gingipain cleavage site for maturation, and β-barrel structure were significantly conserved between the 1439 and Ando strains (Figures 2 and S4).

### Structural characteristics of the purified Mfa1 fimbriae

**Figure 3.**
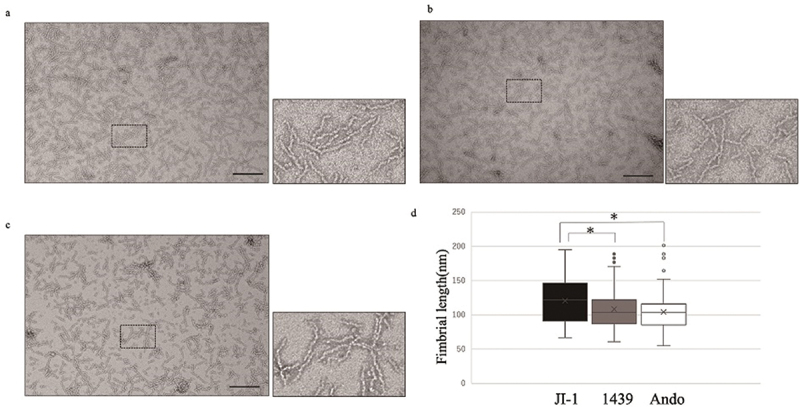
Morphology of the purified Mfa1 fimbriae from three prototype strains. (a) JI-1; (b) 1439; (c) Ando. Mfa1 fimbriae were negatively stained with 2% uranyl acetate. Scale bars = 200 nm. The dashed boxes indicate the magnified regions shown in the lower panels. (d) Comparison of fimbrial length in box-and-whisker plots. One hundred fimbriae were randomly selected, and the fimbrial length was measured using electron microphotographs. * denotes statistically significant differences at *P* < 0.05.

The purified Mfa1 fimbriae of genotypes *mfa1*^*70A*^, *mfa1*^*70B*^, and *mfa1*^*53*^ were analyzed by electron microscopy (Figure 3a–). The length of the purified Mfa1 fimbriae of JI-1 ranged from 60 to 200 nm, consistent with previous observations [40]. Many of the fibers of JI-1, 1439, and Ando were 80–120 nm long, with averages of 121, 108, and 104 nm, respectively. Fimbrial lengths of 1439 and Ando were significantly shorter than those of JI-1 (Figure 3e).

### Variability in antigenic specificity of the Mfa1 protein

**Figure 4.**
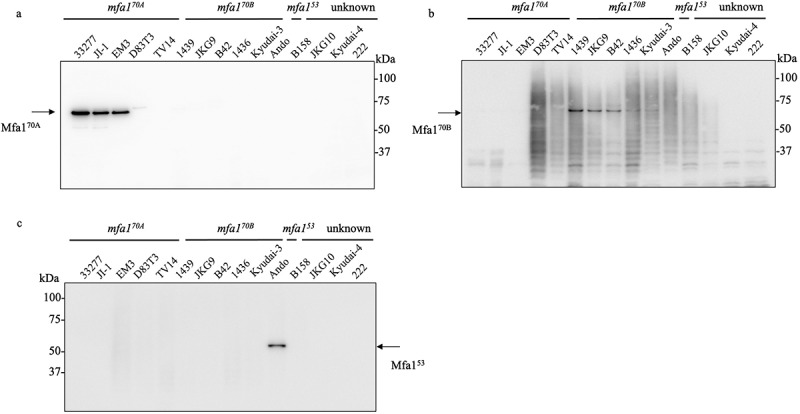
Detection of Mfa1 protein by immunoblot analysis of various Porphyromonas gingivalis strains. Lanes were loaded with 10 µg of WCL from P. gingivalis (a – c). (a) Immunoblot analysis against the Mfa1^70A^ protein. (b) Immunoblot analysis against the Mfa1^70B^ protein. (c) Immunoblot analysis against the Mfa1^53^ protein.

**Figure 5.**
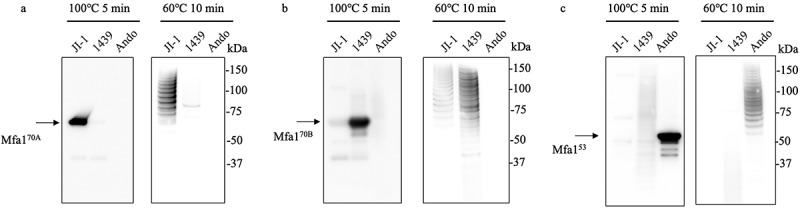
Immunoblot analysis of the purified Mfa1 fimbriae of three prototype strains against Mfa1 protein. Lanes were loaded with 1 µg of pure Mfa1 fimbriae (a – c). (a) Immunoblot analysis against Mfa1^70A^ protein. (b) Immunoblot analysis against Mfa1^70B^ protein. (c) Immunoblot analysis against Mfa1^53^ protein. Samples were denatured at 100°C for 5 min (left panel) or 60°C for 10 min (right panel).

The DNA and amino acid sequences of the prototype strains of *mfa1*^*70A*^ (strain ATCC 33,277) and *mfa1*^*70B*^ (strain 1439) showed 77.2 and 81% similarity, respectively. However, strains with *mfa170B* did not react with antibodies to *mfa170A* in western blot analysis [30], suggesting that there is variability in antigenic specificity between Mfa1^70A^ and Mfa1^70B^. We first performed western blot analysis on the WCL of the 12 strains using an anti-Mfa1^70A^ fimbriae antiserum. Immunoreactive bands were detected for the genotype *mfa1*^*70A*^ in the 33,277, JI-1, EM3, and D83T3 strains (Figure 4a). However, the *mfa1*^*70B*^ strains 1439, JKG9, and B42 did not display anti-Mfa1^70A^ immunoreactive bands, despite distinct Mfa1^70A^ bands detected by CBB staining (Figure S3). This result suggests that Mfa1^70B^ is recognized differently from Mfa1^70A^ in terms of antigenicity. Therefore, we generated rabbit Mfa1^70B^ fimbriae antiserum to verify variability in antigenic specificity among *mfa1* genotypes. Using the anti-Mfa1^70B^ antiserum, western blotting was performed on the WCL from the 12 strains. Distinct 70 kDa immunoreactive bands were detected in 1439, JKG9, B42, 1436, and Kyudai-3 strains (Figure 4b). Finally, the 53 kDa band of Mfa1 was detected in the Ando strain using anti-Mfa1^53^ antiserum (Figure 5c).

Western blotting using anti-Mfa1^70A^, anti-Mfa1^70B^, and anti-Mfa1^53^ fimbriae antisera was performed on purified fimbriae to compare variability in antigenic specificity among genotypes. The results showed that anti-Mfa1^70A^ antiserum specifically detected Mfa1^70A^ protein in purified fimbriae, anti-Mfa1^70B^ antiserum detected Mfa1^70B^ protein, and anti-Mfa1^53^ antiserum detected Mfa1^53^ protein (Figure 5a–). A polymer of Mfa1 protein ladder-like bands was also specifically detected when the purified fimbriae were partially denatured at 60°C (Figure 5a–). However, a weak signal for the polymer of the Mfa1 protein in the JI-1 strain was detected using anti-Mfa1^70B^ antiserum (Figure 5b).

### Cell surface expression of Mfa1 fimbriae in P. gingivalis strains

**Figure 6.**
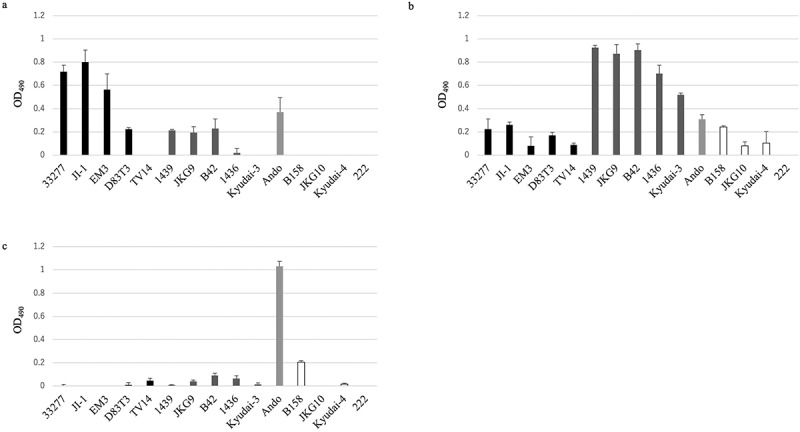
Filtration enzyme-linked immunosorbent assay of intact cells of various Porphyromonas gingivalis strains. P. gingivalis ATCC 33,277, JI-1, EM3, D83T3, TV14, 1439, JKG9, B42, 1436, Kyudai-3, Ando, B158, JKG10, Kyudai-4, 222 and SMF1 strains were applied over filters in a filtration plate at 1 × 10^7^ cells/well. Bacterial cells were probed with antiserum against each genotype of Mfa1^70A^ protein (a), Mfa1^70B^ protein (b), or Mfa1^53^ protein (c), and then with peroxidase-conjugated goat anti-rabbit IgG (1:1000 dilution). Data represent mean absorbance at 490 nm (OD_490_) ± standard deviation (SD) in quadruplicate. Black, dark gray, light gray, and white bars indicate the measurements of mfa1^70A^, mfa1^70B^, mfa1^53^, and unknown genotypes, respectively.

After confirming the variability in antigenic specificity of the antiserum, we analyzed the expression levels of *mfa1*^*70A*^, *mfa1*^*70B*^, and *mfa1*^*53*^ on the surface of the 12 strains using filtration ELISA. The values for the 33,277, JI-1, and EM3 strains (genotype *mfa1*^*70A*^) were significantly higher than those of the strains of *mfa1*^*70B*^ and *mfa1*^*53*^ genotypes when using anti-Mfa1^70A^ antiserum (Figure 6a; Table S1). The expression level in the EM3 strain was as high as that in the 33,277 strain. In contrast, the expression level in the D83T3 strain was significantly lower than that in the 33,277 strain. The values for 1439, JKG9, B42, 1436, and Kyudai-3 (genotype *mfa1*^*70B*^) strains were significantly higher than those for the genotypes *mfa1*^*70A*^ and *mfa1*^*53*^ (Figure 6b; Table S1). Similarly, the values for the Ando strain (genotype *mfa1*^*53*^) were significantly higher than those for the genotypes *mfa1*^*70A*^ and *mfa1*^*70B*^ (Figure 6c; Table S1).

### Variability in antigenic specificity and expression of the minor proteins among P. gingivalis strains

**Figure 7.**
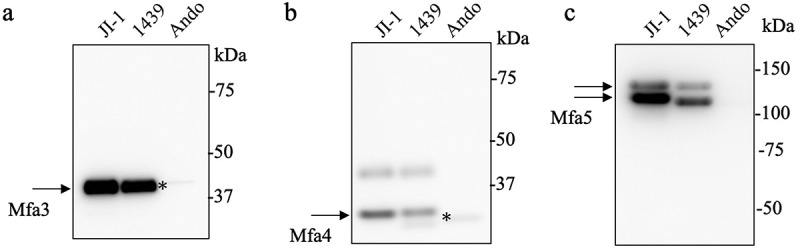
Detection of accessory proteins in the purified Mfa1 fimbriae from three prototype strains. Lanes were loaded with 1 µg of pure Mfa1 fimbriae of different prototype strains (a – c). (a) Immunoblot analysis against Mfa3 (genotype 70) protein. (b) Immunoblot analysis against Mfa4 (genotype 70) protein. (c) Immunoblot analysis against Mfa5 (genotype A1) protein.

In the three different genotypes, including subtypes of fimbriae, the bands of the accessory proteins were detected using CBB staining (Figure 1). We previously reported that *mfa3* and *mfa4* were divided into two genotypes, 70 and 53, and *mfa5* was divided into at least five genotypes, A to E (Figure S2) [31]. The antiserum generated using purified Mfa proteins of the JI-1 strain may distinguish the Mfa proteins between the genotypes with varying efficiency if there are sequence variabilities. To analyze the variability in antigenic specificity of the Mfa3–5 proteins, we first performed western blotting using antiserum against the purified Mfa1 fimbriae samples. The immunoreactive bands of 40-kDa Mfa3 and 30-kDa Mfa4 were clearly detected for the 70 genotype in the JI-1 and 1439 strains, and weak bands were detected for the 53 genotype in the Ando strain (Figure 7a,b). The 130- and 150-kDa immunoreactive bands of Mfa5 were detected in the JI-1 (genotype A1) and 1439 (genotype A2) strains; however, they were below the limit of detection in the Ando strain (unknown genotype because of the lack of sequencing data on *mfa5*) (Figure 7c). No immunoreactive Mfa2 band was detected in the purified fimbriae of 1439, JI-1, or Ando strains (Figure S5).

**Figure 8.**
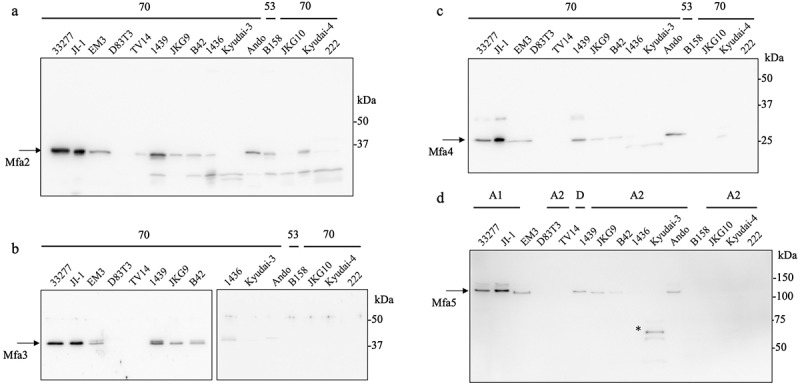
Detection of accessory proteins of various Porphyromonas gingivalis strains. Lanes were loaded with 10 µg (a – c) or 20 µg (d) of WCL. (a) Immunoblot analysis against Mfa2 (genotype 70) protein. (b) Immunoblot analysis against Mfa3 (genotype 70) protein. (c) Immunoblot analysis against Mfa4 (genotype 70) protein. (d) Immunoblot analysis against Mfa5 (genotype A1) protein. A potential degradation product is indicated by an asterisk.

Next, we compared the variability in antigenic specificity and expression of the minor components of the fimbriae using western blotting with the WCL of the 12 strains. Mfa2 was detected in the WCL with strong signals in the 33,277, JI-1, and 1439 strains, whereas weak signals were detected in the EM3, TV14, JKG9, B42, 1436, Ando, B158, and Kyudai-4 strains (Figure 8a). A strong Mfa3 signal was detected in the 33,277, JI-1, and 1439 strains, whereas weak signals were detected in the EM3, JKG9, B42, 1436, and Ando strains (Figure 8b). Mfa4 was detected in the 33,277, JI-1, EM3, 1439, JKG9, B42, and Ando strains (Figure 8c). Mfa5 was detected in the 33,277, JI-1, EM3, 1439, JKG9, B42, and Ando strains, whereas a faint band at low molecular weight was detected in Kyudai-3 (Figure 8d). Overall, in the comparison of detection of Mfa2–5 proteins between *P. gingivalis* strains, among the strains classified as the same genotype by Clustal Omega analysis (Figure S2), the proteins with high similarity were strongly detected, whereas the proteins with low similarity were weak or undetectable by western blotting (Figure 8c). The Mfa1–Mfa5 proteins detected in the WCL of all *P. gingivalis* strains are summarized in Table 1.

## Discussion

In this study, we purified Mfa1 fimbriae from *P. gingivalis* 1439, which expresses the subtype *mfa1*^*70B*^. We then compared the properties of Mfa1 with those of the JI-1 and Ando strains, which express the *mfa1*^*70A*^ and *mfa1*^*53*^ fimbriae genotypes, respectively. The results showed that the composition of minor components, based on the predicted SWISS-MODEL protein structure and N-terminal sequencing analysis, and structural properties of the short fimbriae resembled each other; however, there was variability in antigenic specificity between Mfa1^70A^ and Mfa1^70B^ in sequence variabilities, which may be recognized as different proteins in the immune response. In addition, western blotting and filtration ELISA revealed that several *P. gingivalis* strains, including 1439, expressed Mfa1^70B^ fimbriae. Thus, we propose that *mfa1*^*70B*^ is not a subtype but a novel major genotype of *mfa1* in *P. gingivalis*, which may allow a novel genotype-based classification in *P. gingivalis*. In future studies, classification of the genotypes of *mfa1*^*70A*^, *mfa1*^*70B*^, and *mfa1*^*53*^ will be necessary to investigate the relationship between *P. gingivalis* pathogenicity and *mfa1* genotypes in clinical samples.

Among the 1439 and 33,277 strains, the genotypes of the minor components of *mfa2, mfa3*, and *mfa4* were classified as type 70 (Figure S2 and Table 1). Consistent with the results of genotyping, Mfa2, Mfa3, and Mfa4 protein bands were commonly detected in the 1439 and 33,277/JI-1 strains as determined by western blotting using anti-Mfa2, anti-Mfa3, and anti-Mfa4 (genotype 70) antiserum (Figures 7 and 8). In contrast, the signal intensity of the immunoreactive bands of Mfa3 and Mfa4 in the Ando strain, classified as genotype 53, was considerably weak (Figures 7 and 8). However, the Mfa3 and Mfa4 protein bands were slightly detected in the purified fimbriae (Figure 1). Furthermore, the Mfa1 fimbrial structure of strain Ando also retained their shorter structure than in that of JI-1, indicating that Mfa2, an elongation terminator [11,12], is expressed and functional in the Ando strain. These results suggest that Mfa2–4 proteins are expressed in Ando. Protein expression analysis using anti-Mfa2, Mfa3, and Mfa4 of genotype 53 was not performed in this study; hence, although the exact mechanisms are not clear, the weak signals of the Mfa2, Mfa3, and Mfa4 bands in WCL (Figure 8) suggests variability in antigenic specificity among genotypes 70 and 53, similar to that of Mfa1.

Immunoblot analyses demonstrated differences in the immunoreactivities of antibodies against Mfa1 proteins from different strains of Mfa1^70A^ and Mfa1^70B^ (Figure 2). Therefore, slight sequence differences between the strains of genotype *mfa1*^*70A*^ and *mfa1*^*70B*^ are indicative of antigenic variation in Mfa1 proteins. Furthermore, the Mfa1^70A^ and Mfa1^53^ proteins showed a low amino acid sequence homology and different antigenicity [30]. These results suggest that there are at least three serotypes among Mfa1 fimbriae that are closely associated with the genotypes. However, cross-reactivity was detected between Mfa1^70A^ and Mfa1^70B^ proteins; in particular, Mfa1^70A^ polymers were recognized by anti-Mfa1^70B^ sera (Figure 5b), suggesting that they might express common conformational epitopes in fimbrial structure.

CBB and western blotting analyses revealed that 33,277, EM3, 1439, JKG9, B42, and Ando strongly express the Mfa1 protein. Filtration ELISA using intact cells demonstrated that these strains expressed the protein on the cell surface. However, it was also partly detected among different genotypes and in strains that did not express Mfa1 such as the B158, JKG10, Kyudai-4, and 222 strains, although no significant differences were found between these strains (Figure 6) (Table S1). These results suggest that nonspecific reactions occur to some extent in intact bacteria. Although we did not perform further experiments in the present study, we plan to analyze the structure of fimbriae on the cell surface of *P. gingivalis* strains using electron microscopy and immunoelectron microscopy in the future.

The Mfa5 protein was below the limit of detection in the purified fimbriae from the Ando strain in both SDS-PAGE and western blot analyses. There are two possible reasons for this result. First, Ando showed low expression levels of Mfa5 because of a mutation in *mfa5* [31]. Second, the Mfa5 antigenicity differs because of different *mfa5* genotypes. The cause of this low expression is unclear from this study; however, the low content of Mfa3 and Mfa4 in Ando is consistent with our reported finding that Mfa5 is required for the incorporation of Mfa3 and Mfa4 into fimbriae [13]. Compared to the Ando strain, Mfa5 was detected in the Kyudai-3 strain, whereas the other Mfa2–4 proteins were not detected in the WCL (Figure 8). Moreover, Mfa5 was degraded in the Kyudai-3 strain. These results suggest that Mfa2–4 are involved in the stabilization of Mfa5. However, Mfa5 function, expression, genotype, and antigenicity require further analysis.

Mfa5 structurally differs from the other Mfa proteins [10]. Heidler et al. [41] reported that Mfa5 contains a von Willebrand factor type A domain and structurally resembles streptococcal tip adhesins, such as RrgA and GBS104. In the present study, we showed that Mfa1^70B^ fimbriae also contain Mfa5 (genotype A2) (Figures 1 and 7). Mfa1 fimbriae adhere to the SspB polypeptide on the surface of the oral commensal bacterium *Streptococcus gordonii* [40], and *P. gingivalis* interacts with host cells through Mfa1 fimbriae to elicit inflammatory responses [19,20,42,43]. We found that although the sequencing was incomplete, the EM3 and D83T3 strains possessed two *mfa5* genes (genotypes A2 and E) in tandem, expressing Mfa1 fimbriae on the cell surface [31]. We focused on the functional analysis of Mfa5 in these strains.

The Mfa1 protein was not detected by western blotting in the 222, B158, JKG10, Kyudai-4, or TV14 strains. These results are consistent with the absence of the *mfa1* gene in the 222 strain and the possible mutations in *mfa1* previously reported in the B158, JKG10, Kyudai-4, and TV14 strains [31]. Among the Mfa1–5 proteins, at least one protein was not detected in the 222, 1436, B158, D83T3, JKG10, Kyudai-4, and TV14 strains (Table 1). As Mfa2–5 proteins not only form the fimbrial structure but also influence the biogenesis of fimbriae [10], these *P. gingivalis* strains likely exhibit various Mfa1 fimbrial structures with different components and lengths. Mutations in the *fim* and *mfa* clusters are even found in wild-type strains worldwide. For example, W83 is an afimbrial strain due to inactivation caused by transposon insertions in *mfa1* and *fimA* [44]. The ATCC 33,277 strain expresses a nonsense mutation in the *fimB* gene immediately downstream of *fimA* and produces abnormally long fimbriae lacking functional FimB, which is an anchor protein with the same function as Mfa2 [45,46]. Taken together with our genotype sequencing and antigenic analyses of Mfa1 proteins, we revealed that the *mfa* clusters might contain global clonal variations of the genotypes responsible for differential antigenicity and fimbrial structure among *P. gingivalis* strains. Clonal variations in *P. gingivalis* are related to the bacterial infectious traits that influence periodontal and systemic diseases [21]. In the future, pathogenic and functional analyses based on the genotype of the proteins, including the fimbrial structure in *P. gingivalis*, should be performed.

## Supplementary Material

Supplemental Material
