# Supplementary material for: Structural and antigenic characterization of a novel genotype of Mfa1 fimbriae in Porphyromonas gingivalis
Source: J Oral Microbiol. 2023 May 21;15(1):2215551. doi: 10.1080/20002297.2023.2215551 (PMC10201998; doi:10.1080/20002297.2023.2215551)
Supplement: Supplemental Material [file ZJOM_A_2215551_SM9112.zip › Supplementary files/Supplementary material.docx]

**Supplementary material**

**Figure S1. The *mfa* gene cluster and predicted structure of Mfa1 fimbriae.**

(a) Gene map of *mfa* clusters encoding fimbrial proteins on the chromosome. The cluster contains five genes with the same transcriptional direction. Sequences were derived from NCBI accession NC_010729 (*P. gingivalis* ATCC 33277, complete genome) [46]. (b) Schematic diagram of the potential structural model of *P. gingivalis* fimbriae. Mfa1 fimbriae are composed of Mfa1 polymers associated with Mfa3–5 accessory proteins at the tip. Outer membrane Mfa2 proteins associate with Mfa1 filaments at the base. The sequence order of the tip proteins is unknown.

**Figure S2. Similarities among amino acid sequences of Mfa proteins from various *Porphyromonas gingialis* strains as per the algorithms in the Clustal Omega multiple alignment tool.** The numbers shown indicate the Omega score. The *mfa1* genes indicated by M and X in Table 1 were excluded from the cluster Omega analysis.

**Figure S3. Sodium dodecyl sulfate-polyacrylamide gel electrophoresis (SDS-PAGE) and Coomassie Brilliant Blue (CBB) staining of the whole cell lysates (WCL) of various *Porphyromonas gingivalis* strains.** Lanes were loaded with 20 µg of WCL. Asterisks (*) indicate Mfa1 protein bands.

**Figure S4. 3D-SWISS-model of Mfa1 proteins.**

The amino acid sequences encoding Mfa1 from the ATCC 33277 (a), 1439 (b), and Ando (c) strains were subjected to SWISS-MODEL analyses. Homology modeling was performed using Mfa1 from ATCC 33277 (5nf3.1. A in PDB) as a template. The quality of the protein structural models is indicated by qualitative model energy analysis (QMEAN); blue and red indicate high and low specific feature quality scores, respectively. The structural model of the Mfa1^70B^ protein from the 1439 *P. gingivalis* strain showed very high concordance with the Mfa1^70A^ protein from the ATCC 33277 strain (PDB code: 5dhm). The Mfa1^53^ protein from the Ando strain displayed low overall QMEAN values; however, the β1β2-loop at the Arg^49^ gingipain cleavage site, β-barrel structure, and structure determination were significantly conserved.

**Figure S5. Immunoblot analysis of the purified Mfa1 fimbriae of prototype strains against the Mfa2 (genotype 70) protein.** 33277 WCL: Positive control for Mfa2 proteins. No immunoreactive bands were detected in the purified fimbriae from three prototype strains.
