# Supplementary figures and images for: Structural and antigenic characterization of a novel genotype of Mfa1 fimbriae in Porphyromonas gingivalis
Source: J Oral Microbiol. 2023 May 21;15(1):2215551. doi: 10.1080/20002297.2023.2215551 (PMC10201998; doi:10.1080/20002297.2023.2215551)

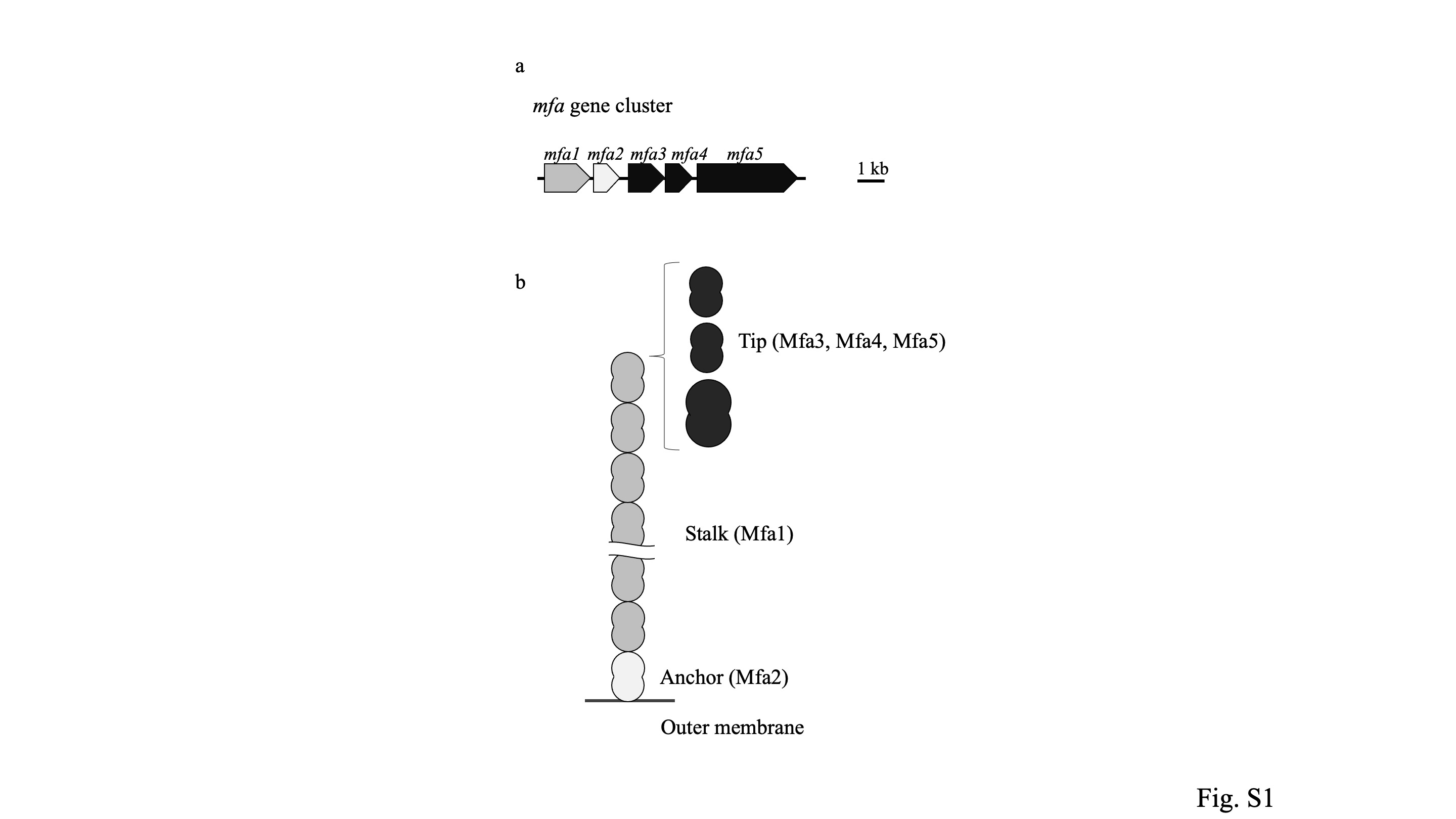

Supplement: Supplemental Material [file ZJOM_A_2215551_SM9112.zip › Supplementary files/ZJOM20220084 Supplement Fig1.jpeg]

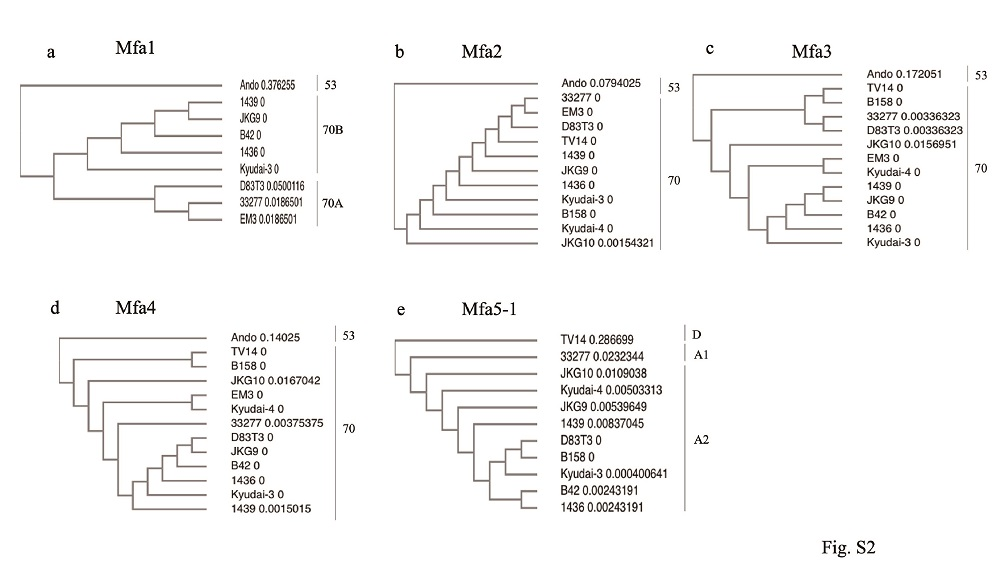

Supplement: Supplemental Material [file ZJOM_A_2215551_SM9112.zip › Supplementary files/ZJOM20220084 Supplement Fig2.png]

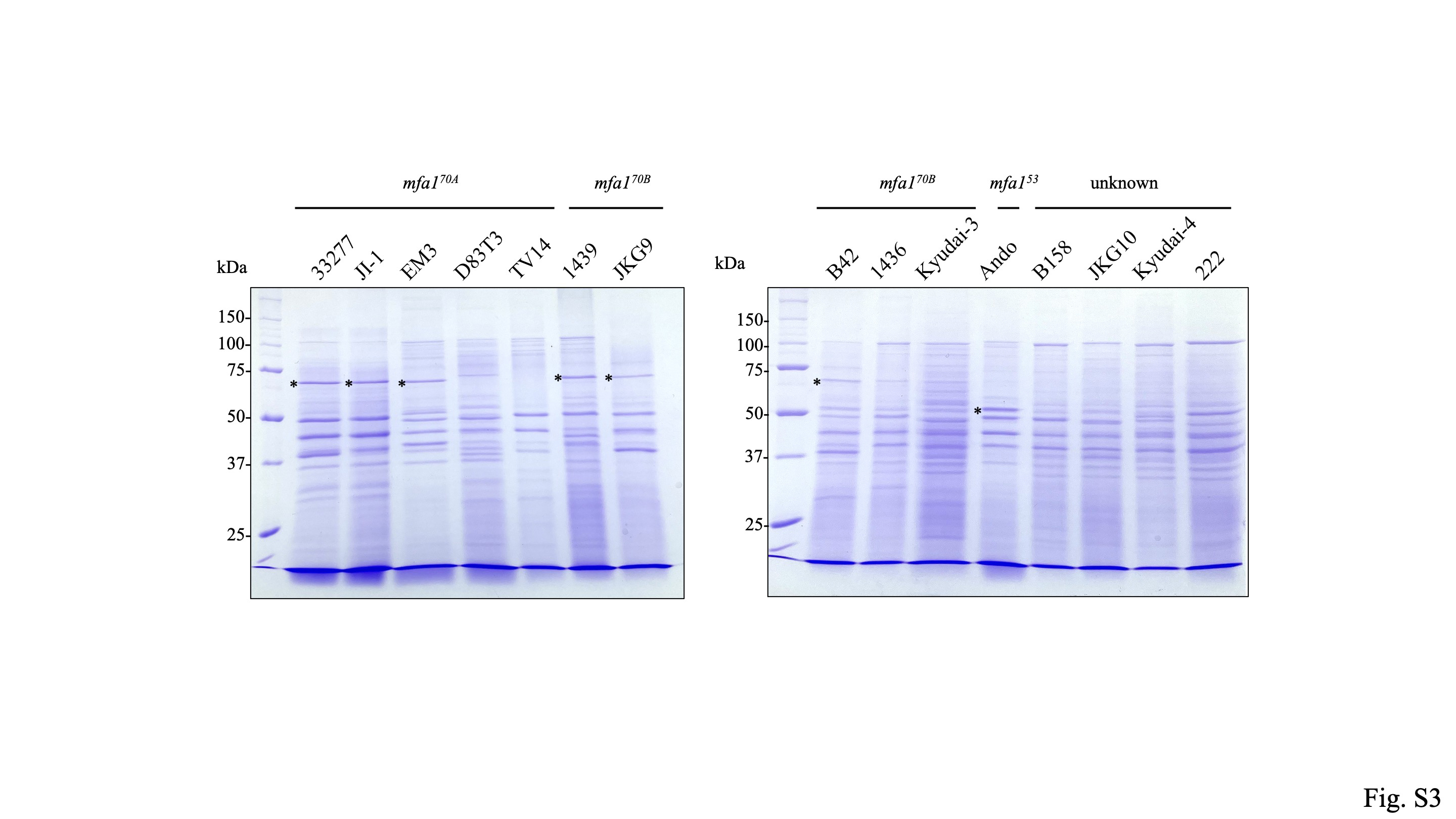

Supplement: Supplemental Material [file ZJOM_A_2215551_SM9112.zip › Supplementary files/ZJOM20220084 Supplement Fig3.png]

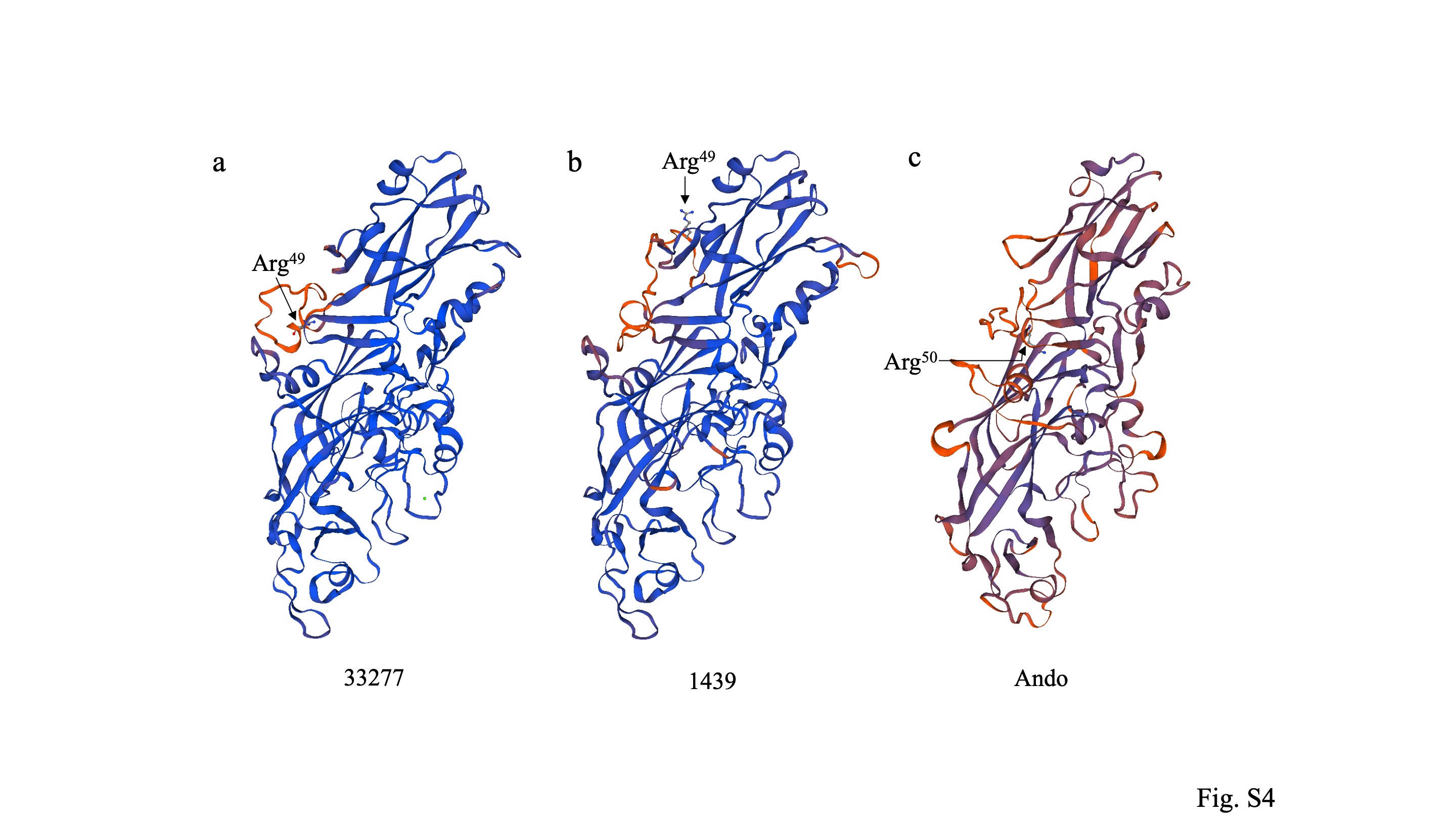

Supplement: Supplemental Material [file ZJOM_A_2215551_SM9112.zip › Supplementary files/ZJOM20220084 Supplement Fig4.jpeg]

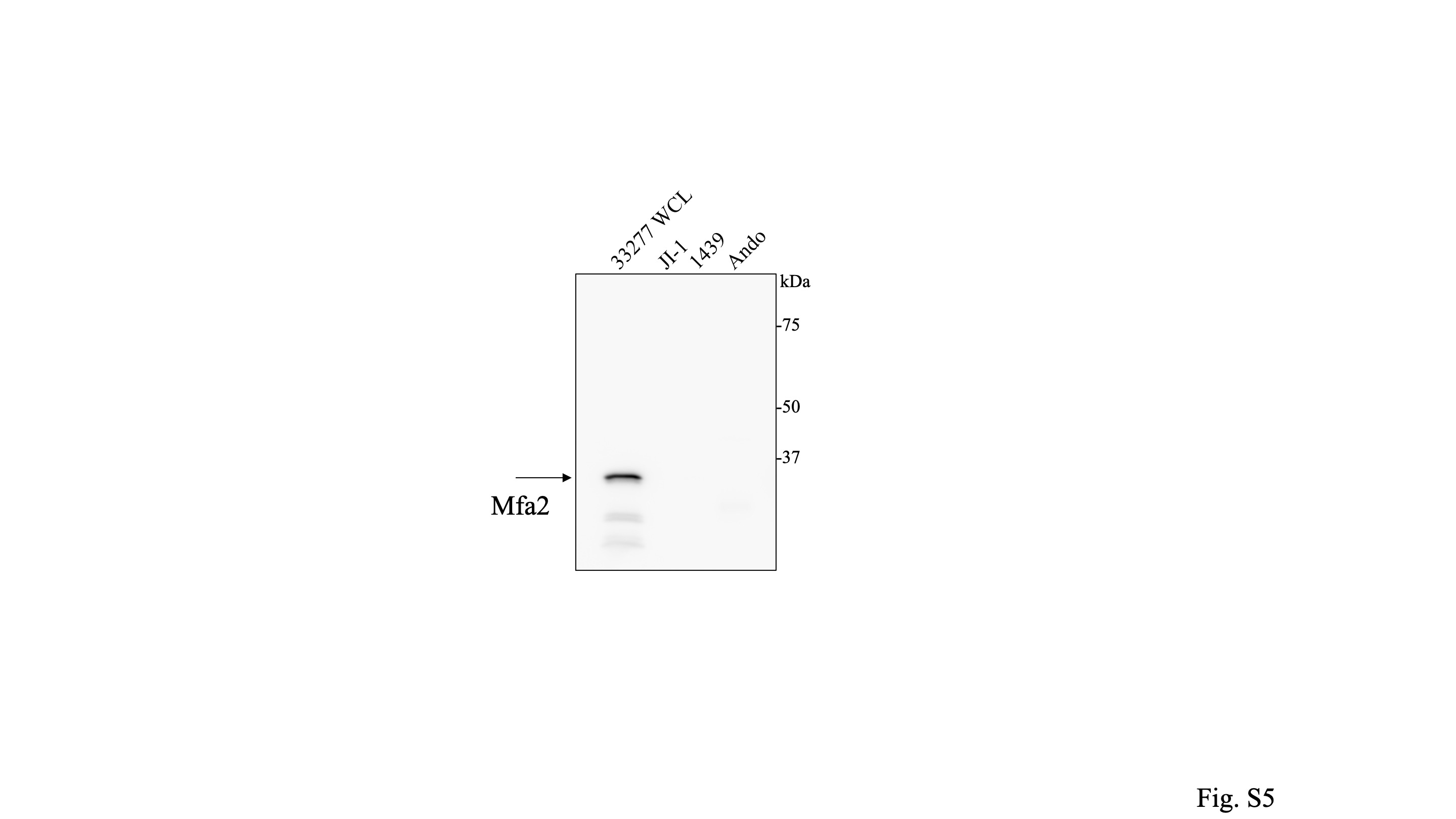

Supplement: Supplemental Material [file ZJOM_A_2215551_SM9112.zip › Supplementary files/ZJOM20220084 Supplement Fig5.jpeg]
